# “A Streetcar Named Instagram Desire”: Evolutionary Psychological Perspectives on the Multifarious Human Desires That Shape Instagram Selfie-and-Groupfie Cultures

**DOI:** 10.3390/bs12100396

**Published:** 2022-10-17

**Authors:** S. Venus Jin, Ehri Ryu

**Affiliations:** 1 NU-Q Communication Program, Northwestern University in Qatar, Doha 34102, Qatar; 2 Department of Psychology, Boston College, Chestnut Hill, MA 02467, USA

**Keywords:** Instagram culture, social media, social networking sites, structural equation modeling, selfies and groupfies, evolutionary psychology

## Abstract

Instagram not only offers an arena for the fulfillment of basic human desires but also cultivates new types of multifaceted desires and consumptions in Web 2.0 environments. This study aims to examine a wide variety of dispositional, psychological, and attitudinal predictors of Instagram consumption and selfie-and-groupfie cultures. Three cross-sectional surveys (Study 1 (*N* = 108); Study 2 (*N* = 140); Study 3 (*N* = 557)) were conducted, and empirical data were analyzed using structural equation modeling (SEM) with Mplus 8.0. Study 1 shows associations among appearance-related self-confidence, appearance-related actual–ideal self-discrepancy, materialism, and Instagram consumption. Study 2 confirms relationships among weight status perception, self-esteem, eating disorder, malicious envy, and Instagram consumption intensity. Study 3 further demonstrates dynamic associations among eating disorders, perceived mate value, narcissistic grandiosity, envy, social comparison, intrasexual competition for mates, and frequency of posting selfies/groupfies on Instagram. Theoretical contributions to the psychosocial and human aspects of the Web 2.0 digital culture, managerial implications for online dating cultures, and practical implications for consumption markets including social media-based health communication, cultural communication, and marketing communication are discussed.

## 1. Introduction

### 1.1. Instagram

Instagram, as one of the most popular social networking sites (SNSs) worldwide, is a mobile social media platform that enables users to edit and share photos and videos. The number of monthly active users (MAU) of Instagram, owned by Facebook, reached 1 billion in 2018, up from 800 million in 2017 [1]. As of 28 January 2022, the daily active users (DAU) stood at 500 million globally [2,3]. 

There is abundant literature on the effects of using various SNSs on a wide range of endogenous variables including social [4], psychological [5,6,7], and behavioral [8] outcomes. Thus, the extant literature mostly answers “how” social media technology influences human psychology and “what” effects social media platforms and contents have on human cognition, emotion, and behavior. Most recently, for example, a Wall Street journal investigation [9] prompted a fierce discourse about the detrimental effects of Instagram on teen girls’ body image and mental-health issues [10]. 

### 1.2. Why Do People Use Instagram?

However, relatively little attention has been paid to exogenous variables and antecedent factors that predict Instagram usage intensity, thus disregarding the “why” question. If people know that Instagram has negative effects such as anxiety, depression, and body image issues [11], why do they still use the app? The main research question (RQ) of the current research, therefore, is: “Why do people use Instagram and what types of people use Instagram more frequently?” Furthermore, there is a dearth of theoretical discussion about the psychological mechanisms that explain the sequential processes consisting of users’ dispositional factors (personality and individual differences), fundamental desires (basic human needs), and actual Instagram usage metrics (overall platform usage and actual content-posting behaviors). To address this research gap, the present study attempts to examine multifarious personality factors, individual difference factors, and human desires as predictors of Instagram form usage and content usage behaviors, such as narcissism, self-discrepancy, social comparison, materialism, eating disorder, intrasexual competition for mates, and perceived mate value. Two dimensions of Instagram form and content usage as endogenous variables were examined: (1) subjective perception of Instagram usage intensity and (2) objective and quantitative Instagram usage indexes such as frequency of platform usage, selfie-posting intention/behavior, and groupfie-posting intention/behavior. The cultural fascination with selfies, as social media forms of self-portraiture, has been explored from the various lenses of critical cultural theories including feminist representational politics [12] and journalistic, clinical, and ideological discourses on narcissism [13] in the consumption markets and culture literature.

### 1.3. Human Desires Fulfilled and Cultivated through Instagram

Instagram not only offers an arena for the fulfillment of basic human desires but also creates and cultivates new types of multifaceted desires in Web 2.0 environments. Instagram’s popularity and exponential growth are partially attributed to the fulfillment of basic social needs (e.g., need to connect, need for affection, and need to belong) [14,15]. Instagram also has cultivated and ignited burning desires such as desires to approve/follow/be followed by others online [16], selectively present filtered photos and selfies [17], digitally showcase successful lifestyles [18], and strategically show off material possessions [19]. The influx of Instagram fashionistas’ stylish photos featuring high fashion items [20], foodies’ photos featuring gourmet foods and fine dining experiences [21], super-rich kids’ conspicuous consumption and presentation of luxury possessions symbolizing a lavish lifestyle [18], and popular social media celebrities’ glamorous photos featuring attractive body images [22] as well as the digitized fashion market transformed through hashtagged visual and textual brand–consumer conversations on Instagram [23] represent the manifestation of multifaceted desires of Instagram photo posters. Furthermore, these conspicuous exhibitions and strategic fulfillments of photo posters’ desires are collectively acknowledged and socially approved by peer Instagram viewers, as quantitatively indexed by millions of followers and likes [17]. These desires cultivated via Instagram and other visual social media with popularity cues correspond to humans’ social and egoistic needs (e.g., desire to be publicly acknowledged by others and socially approve others in the form of liking/following/commenting, need for prestige, success, accomplishment, self-esteem, and popularity). 

### 1.4. Research Objectives and Key Research Questions (RQs)

Providing deeper insights into the fundamental human desires that drive Instagram usage behaviors is the impetus for conducting the current research consisting of three cross-sectional surveys. To achieve the research objective, this study elaborates on the relevance of users’ materialistic values, body image perception, and narcissism to the essence of Instagram, drawing upon the theoretical underpinnings elaborated in the following literature review. Ultimately, the current research aims to answer the three key research questions (RQs): (1) “Why do people use Instagram?” (2) “What types of people use Instagram more frequently?” (3) “What are dispositional, psychological, and attitudinal predictors of Instagram consumption and selfie-and-groupfie posting behaviors?” In order to answer these main RQs, the current study consists of three cross-sectional surveys. Study 1 focuses on self-perception and materialism as antecedents of Instagram usage. Building upon the preliminary findings from Study 1, Study 2 focuses on self-perception, eating disorder, and materialistic envy as antecedents of Instagram usage. Building upon the findings of Study 1 and Study 2, Study 3 presents the results of testing more sophisticated and integrative models that propose self-perception, perceived mating value, intrasexual competition for mates, narcissism, materialism, eating disorder, and social comparison as antecedents of selfie-and-groupfie-posting behaviors. 

## 2. Theoretical Background

### 2.1. Study 1: Materialism and Instagram

#### 2.1.1. Materialism, Envy, and Instagram

Materialism is defined as “a set of centrally held beliefs about the importance of possessions in one’s life” [24] (p. 308). The consumption of conspicuous goods and display of these material possessions via electronic word of mouth (eWoM) influence the intensity of using social media [25,26] due to users’ desire to show off acquired goods and share them with their social network. Desire to display material possessions and experiential purchases on social media [19,27] partially accounts for the popularity of Instagram among consumers who post and share photos featuring their acquired luxury goods and show off their conspicuous consumption symbolizing a lavish lifestyle. Envy, referring to “an unpleasant and often painful blend of feelings caused by a comparison with a person or groups of persons who possess something we desire” [28] (p. 49), is positively associated with materialism [29] and materialistic display on social media [27]. Envy, as one dimension of materialism [29], is generated when others possess envied products or brands and is prevalent on Instagram [19]. No prior research examined envy and materialism as predictors of Instagram usage intensity. In light of Instagram’s affordability to satisfy users’ desires to show off material possessions [20], display taste and luxurious lifestyles [18], and exhibit attractive face and body [22,30] as well as Instagram’s symbolic function as an arena for conspicuous display, social comparison, and consequent envy [19], Study 1 addresses associations among body image perception, materialism, envy, and Instagram usage intensity. 

#### 2.1.2. Body Image Perception, Materialism, and Instagram 

Self-schema refers to “cognitive generalizations about the self, derived from past experience, that organize and guide the processing of self-related information contained in an individual’s social experience” [31] (p. 64). Technology users who have higher self-esteem have lower levels of appearance self-discrepancy [32]. Appearance schema represents a cognitive component of body image [33]. Self-schema and self-discrepancy mediate the influence of Instagram usage on body image satisfaction among youth [32]. Building upon the relevance of self-discrepancy to body image perception in the context of Instagram, Study 1 proposes that appearance-related self-confidence and self-discrepancy predict Instagram usage intensity via materialistic values. Self-confidence has been extensively tested in body image studies about the influence of media exposure on body satisfaction [34,35] and social comparison theories of body dissatisfaction [36], thus implying its relevance to body image perception in response to images posted on social media that stimulate social comparison and induce envy. Self-discrepancy is conceptually defined as the extent to which one’s “actual self” deviates from one’s “ideal self” [37]. Study 1 examines the physical attractiveness aspect of actual–ideal self-discrepancy, which is operationally defined as the discrepancy between an individual’s perceived physical attractiveness of the actual self and that of the ideal self. 

Study 1 further proposes a theoretical model that links appearance-related self-confidence and self-discrepancy via multiple dimensions of materialism to Instagram usage intensity. Richins [38] defined materialism as a set of centrally held beliefs about the importance of possessions in life and delineated three dimensions of materialistic values: success, acquisition, and happiness. Materialists believe that material acquisitions are a symbol of accomplishments and success, and that money is an integral component of happiness. Belk [29] viewed materialism as a set of personality traits consisting of possessiveness, nongenerosity, and envy dimensions. Possessiveness refers to the fear of an individual for losing self-possessions, nongenerosity refers to an individual’s unwillingness to share self-possessions with others, and envy refers to the discontentment of an individual on others’ success and happiness [29]. These multiple dimensions of materialism may be associated with Instagram users’ self-concept as explicated drawing from self-discrepancy theory [37] and the compensatory consumer behavior model [39]. Material acquisition and possession, as compensatory behaviors, increase as a function of discrepancies between how individuals see themselves (actual self) and how they would like to be (ideal self) [39,40]. Actual–ideal self-discrepancy can be a motivator of compensatory behavior such as material acquisition aimed at reducing the perceived gap between the actual self and the ideal self [37,39,40]. Materialistic values direct an individual toward consumption as a strategy to deal with perceived discrepancies in the self-concept [40]. The insecurity-based view on materialism suggests that materialists compensate for concerns about their self-worth and feel secure through material possession [41]. This view describes materialists as individuals with low self-confidence [42], such that individuals with fear of insecurity, high self-discrepancy, and low self-esteem are more likely to be materialistic [43]. Li et al. [44] empirically showed that materialism could compensate for low self-esteem. In contrast, the opposing view depicts materialists as assertive owners of expensive goods and avaricious holders of substantial amounts of money, which implies a positive correlation between materialistic values and high self-confidence [41]. Based on these theoretical rationales and in light of the dual nature of materialism (two opposing possibilities of insecurity-based view versus confidence-based view on materialists), H1 attempts to examine relationships among self-confidence, self-discrepancy, and materialistic values.

**H1:**  *Instagram users’ (a) self-confidence and (b) self-discrepancy are predictors of materialism*.

Materialistic values influence a wide range of beliefs, attitudes, and behaviors [41]. There is a positive correlation between social media intensity and conspicuous consumption [26]. Compulsive social media users are likely to experience low self-esteem and are more likely to turn to social media outlets to feel better [45]. Recent experimental research posits a causal relationship between materialistic values and Facebook usage and empirically proves that high materialistic concerns increase Facebook activity [25]. Although Okazaki et al. [46] attempted to test the moderating role of materialism on compulsive Instagram use, no significant effect of materialism was found. To address this gap, H2 proposes an association between materialism and Instagram usage. It can be hypothesized that materialistic people are more likely to be attracted to Instagram, which is an arena for conspicuous exhibition of material goods and lifestyles [18,19,20], thus resulting in heavier usage of Instagram.

**H2:**  *Instagram users’ materialism is a predictor of (a) quantitative and objective Instagram usage frequency and (b) subjective perception of Instagram usage intensity*.

**Figure 1:**
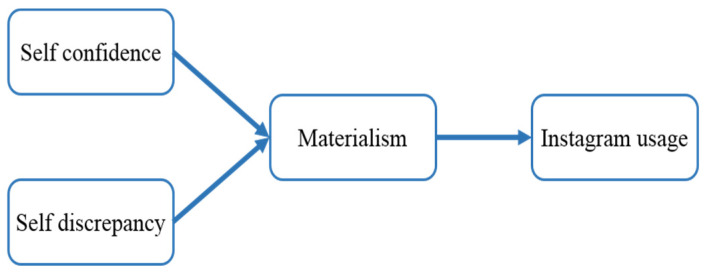
Study 1: Conceptual model.

Figure 1 presents a conceptual and theoretical model that integrates these local level hypotheses proposed in the first cross-sectional data collection through Study 1.

### 2.2. Study 2: Eating Disorder and Instagram

#### 2.2.1. Body Image Perception, Eating Disorder, and Instagram

Instagram is inherently a visual image-based platform, which makes it an appearance-focused environment [10,47]. People with higher levels of body image concern may be attracted to appearance-focused activities on social media [48,49,50]. In light of the influx of body-related visual images and body talk posted on a variety of SNSs, there have been numerous studies about the impact of social media on body image, including the effects of Instagram images on mood [5], the effects of fitspiration and thinspiration images on body image perception [51,52], the impact of SNSs on disordered eating [8], and social comparison and appearance comparison made through social media [6,53,54]. Prior research also examined associations between Facebook use and body image concerns [7], the influence of appearance comparison in social media on body dissatisfaction [6], the effect of attractive Instagram images on mood [5], proliferation of thinspiration images on Twitter, Pinterest [51], and Instagram [22], relationship between SNSs and eating disorder [8], and the influence of psychological distress on social media use [55,56]. Thus, a growing body of research has examined these contemporary issues revolving around the theme of exposure to social media and its impact on body image perception [22,54] and eating disorders [8], using various research methodologies, including cross-sectional survey [7], longitudinal study [57], experiment [5,22,48], content analysis [51], meta-analysis [8], and qualitative interview [56]. However, there is a dearth of structural equation modeling (SEM) research that collected cross-sectional data to examine people’s subjective weight status perception, general self-esteem, eating disorder, and malicious/benign envy as antecedents that may predict Instagram usage. Study 2 aims to address this gap. 

Instagram usage has been linked to a wide range of body image concerns [48]. For example, Instagram use causes appearance comparison and lower body satisfaction [58]. Eating disorders, which consist of multiple dimensions including perfectionism, anorexia, and bulimia nervosa, are psychophysiological illnesses that can lead to serious health consequences [55]. The extant literature theoretically posits that perceived weight status and self-esteem are significant predictors of eating disorder [59] and empirically shows that there is a negative association between self-esteem and perceived weight status perception (thin to obese) [59]. Body dissatisfaction is the strongest and most consistent predictor of disordered eating and clinical eating disorders [60,61]. No prior research has particularly examined Instagram users’ self-esteem and weight status perception as predictors of their eating disorder. Theoretical foundations and empirical evidence discussed so far guided the formation of H1a and H1b:

**H1a:**  *Instagram users’ self-esteem is a negative predictor of multiple dimensions of eating disorders (perfectionism, anorexia, and bulimia nervosa)*.

**H1b:**  *Instagram users’ weight status perception (thin to obese) is a positive predictor of multiple dimensions of eating disorder (perfectionism, anorexia, and bulimia nervosa)*.

#### 2.2.2. Eating Disorder, Envy, and Instagram

Instagram usage frequency predicts visual attention to high-anxiety body regions in young women [62]. The overall time spent on SNSs is related to indexes of body image perception and disordered eating [8]. Maladaptive patterns of social media use, such as excessive reassurance seeking, are associated with body dissatisfaction and disordered eating [63]. For example, empirical research shows that there is a positive correlation between time spent on Facebook and disordered eating [64]. However, no prior research proposed a theoretical model that links multiple dimensions of eating disorder via envy to Instagram usage intensity. To fill this gap in research, Study 2 further examines the dynamic relationships among these concepts. 

Benign envy is characterized by a positive attitude toward the envied person and a desire to improve one’s own inferior position, whereas malicious envy is characterized by hostile feelings and a tendency to destroy the superior position of the envied person [65]. Benign envy prompts people to focus on the means to improve oneself, whereas malicious envy drives people to pay more attention toward the envied person [27]. Benign envy and malicious envy are prevalent on social media, as browsing others’ social media accounts evokes envy [19,27]. Envy mediates the link between social comparison and appearance enhancement [66]. Multiple dimensions of eating disorder (perfectionism, anorexia, and bulimia nervosa) are associated with benign envy and malicious envy prevalent on Instagram [21]. Furthermore, envy evoked on social media mediates the effects of social media posts on users’ psychological response to photo posters [21]. Departing from prior research on the causal effects of Instagram posts on envy [21,27], Study 2 proposes eating disorders as a predictor of envy (H2) and envy as a predictor of Instagram usage (H3).

**H2:**  *Instagram users’ multiple dimensions of eating disorders (perfectionism, anorexia, and bulimia nervosa) are predictors of benign envy and malicious envy*.

**H3:**  *Instagram users’ envy is a predictor of (a) quantitative and objective Instagram usage frequency and (b) subjective perception of Instagram usage intensity*.

**Figure 2:**
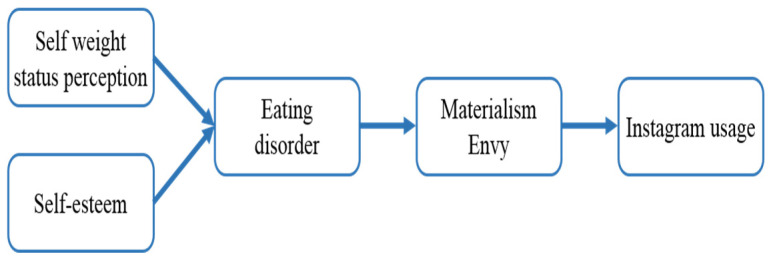
Study 2: Conceptual model.

Figure 2 presents a conceptual and theoretical model that integrates these local-level hypotheses proposed in the second cross-sectional data collection through Study 2.

### 2.3. Study 3: Body Image Perception, Perceived Mate Value, and Narcissism

#### 2.3.1. Instagram Selfies and Narcissism

As another quantitative indicator of Instagram usage, Study 3 measured users’ selfie/groupfie posting frequency and selfie/groupfie upload counts. Selfies refer to self-portraits that a person takes using a smartphone or webcam [67], and groupfies refer to group selfies [68]. Across disciplines, recent studies examined the psychological effects of posting SNS selfies/groupfies [68], the effects of selfies on self-esteem and social sensitivity [69], romantic selfie-posting behavior and love levels [70], selfie-editing frequency and social comparison [67], marketing implications of consumers’ selfie-taking [71], association between narcissism and selfies [72,73], relationship between social exhibitionism and frequency of selfie-posting [74], and more. Veldhuis et al. [49] suggest that body image not only serves as an outcome of selfie-behaviors but also as a motive preceding selfie-behaviors. Despite the exponential increase in research on selfies/groupfies posted on social media and the relevance of selfies/groupfies to body image perception and narcissism, no previous study has provided in-depth theoretical discussions about psychological mechanisms that explain the dynamic relationships among body image, eating disorders, narcissism, evolutionarily adaptive mechanisms of mating efforts, and Instagram selfie/groupfie posting frequency. Study 3 addresses this gap, drawing from the literature on body image and eating disorder as well as evolutionary psychology of narcissism.

Nonpathological narcissism has been associated with social media addiction [75]. Narcissism is a relatively stable individual feature encompassing grandiosity, self-love, a sense of specialness, and inflated self-views [76]. Evolutionary psychology-driven research shows narcissism is associated with mating efforts [77]. As symbolized by the original Greek myth of beautiful Narcissus, there may be a positive correlation between physical attractiveness and narcissism. According to evolutionary psychology, enhancing and displaying one’s own physical attractiveness can assist mate choice [77]. Grandiose beliefs about narcissists’ own physical attractiveness may be the underlying cause of self-absorption and public display of face and body in a variety of social media channels [15,17], which serves as theoretical rationales for H1a. From an evolutionary perspective, the conspicuous display of one’s own physical attributes provides an indicator of health, and potential mates attribute higher mate value to those individuals who conspicuously exhibit signals of healthiness [30]. Therefore, it can be hypothesized that there is an association between self-perceived physical attractiveness and self-perceived mate value [78], which rationalizes H1b.

**H1:**  *Instagram users’ self-perceived physical attractiveness is a positive predictor of (a) narcissistic grandiosity and (b) self-perceived mating value*.

#### 2.3.2. Eating Disorders, Narcissism, Envy, and Physical Appearance Social Comparison

Instagram is a relevant context in which users’ physical appearance social comparison can be examined in relation to body image concerns [16]. As narcissism drives an individual to improve self-worth through the enhancement of physical appearance [79], eating disorders may be associated with narcissistic personality (H2a). Vulnerable narcissists’ strong drive to achieve a sense of self-worth through physical grooming leads them to resort to eating disorder behaviors such as dieting and excessive weight concerns. Since there is already abundant literature on the relationship between vulnerable narcissism and eating disorder [79], Study 3 particularly focuses on grandiose narcissism. Grandiose narcissists tend to place excessive attention on their physical appearance, which may be associated with disordered eating tendencies [80]. Narcissism is positively associated with perfectionism [81], which is one dimension of eating disorder. Prior research also shows that anorexia and bulimia nervosa are high among people who are perfectionists and competitive [82], which suggests an association between eating disorder and envy (H2b). Since materialism is significantly and positively correlated with narcissism [41], narcissism is correlated with envy, which is one dimension of materialism [29]. Envy occurs in the process of upward social comparison [28,83]. People with disordered eating behaviors tend to show a high level of social comparison [84], which provides theoretical rationales for H2c. Appearance comparison refers to the process by which people evaluate themselves by comparing their appearance to others [85]. There is cross-sectional evidence for appearance comparison (i.e., basing one’s self-worth on weight/shape in comparison to others) as a correlate of unhealthy weight control behaviors [86]. Thus, appearance-contingent self-worth is associated with disordered eating [87], which justifies H2d. Yellowlees et al. [50] investigated the association between selfie-behaviors and eating disorder symptom severity in a sample of females with clinically severe eating disorder symptoms. Physical-appearance comparison is related to disordered eating [88]. The current research attempts to examine the role played by eating disorders in relation to narcissism and physical appearance social comparison among nonclinical Instagram users. These theoretical frameworks guided the formation of H2.

**H2:**  *Instagram users’ eating disorder (perfectionism, anorexia, and bulimia) tendencies are positive predictors of (a) narcissistic grandiosity, (b) envy, (c) social comparison, and (d) physical appearance comparison*.

#### 2.3.3. Narcissistic Grandiosity, Envy, Social Comparison, and Intrasexual Competition

Envy, referring to an unpleasant emotion that arises from upward social comparisons [89], is associated with narcissism and state self-esteem instability [90]. Grandiose narcissists tend to score high on competitiveness [91] and social comparison orientation [92], both of which are associated with envy (H3a and H4a). Grandiose narcissism is correlated with envious reactions toward superior others in the process of upward social comparison [93]. Thus, there is a positive correlation between grandiose narcissists’ envy and social comparison (H4a and H4b) [94]. Furthermore, upward social comparison plays an important role in narcissistic self-enhancement such as enhancing physical appearance and grandiosely exhibiting physical attractiveness. Narcissistic grandiosity and envy are correlated with comparison propensity and physical appearance comparison [90]. These theoretical foundations rationalize the proposed hypotheses about the dynamic association among narcissistic grandiosity, envy, social comparison, and physical appearance comparison.

An evolutionary biology perspective views narcissism as a strategy of short-term seduction through high physical attractiveness, exhibitionist charm, and interpersonal skills [82]. Adaptationist evolutionary psychology also claims that narcissism emerged as a variation of trait dominance in mating and competition for mates [95]. The level of narcissism can be viewed as the degree of proclivity to pursue mating and adopting a mode of interaction based on seduction rather than empathy [82]. Gilbert, Price, and Allan [96] emphasized the significance of the social comparison process [97] to intrasexual competition, which refers to competition with members of the same sex for access to mates. Intrasexual competition for mates is making oneself more physically attractive to opposite-sex others or making same-sex rivals less appealing to the target opposite-sex other pursued [98]. Intrasexual competition for mates is derived from the Darwinian theory of sexual selection, referring to an underlying mechanism of evolution that explains how males and females have developed strategies to attract and retain high-quality mates [99,100]. One key strategy in intrasexual competition for mates is self-promotion, which refers to the enhancement and display of characteristics such as physical attractiveness to improve one’s ability to compete against rivals [101]. Therefore, narcissistic grandiosity, manifested by grandiose exhibitionism, is evolutionarily adaptive [77]. Jin and Ryu [15] not only theoretically propose that modern technology users’ grandiose narcissism reflects evolutionarily hardwired intrasexual competition for potential mates but also empirically demonstrate that narcissism is a positive antecedent of intrasexual competition for mates. Hendrickse, Arpan, Clayton, and Ridgway [100] found a significant and positive relationship between intrasexual competition for mates and appearance-related comparisons on Instagram. These theoretical propositions and empirical findings drawing from evolutionary psychology of narcissism serve as foundations for H3b and H4c. Thus, Study 3 proposes an integrative theoretical model that links narcissistic grandiosity via envy to social comparison propensity, physical appearance comparison, and intrasexual competition for mates by testing H3 and H4:

**H3:**  *Instagram users’ narcissistic grandiosity is a positive predictor of (a) envy and (b) intrasexual competition for mates*.

**H4:**  *Instagram users’ envy is a positive predictor of (a) social comparison, (b) physical appearance comparison, and (c) intrasexual competition for mates*.

#### 2.3.4. Physical Appearance Comparison, Intrasexual Competition, and Selfies/Groupfies 

Desire for ideal online self-presentation is a motivational factor that prompts social media users to engage in physical appearance social comparison and digital self-enhancement of physical attractiveness such as idealized selfie-posting and selfie-editing [67]. Narcissists are more likely to post self-promoting content, specifically, self-focused pictures or selfies, compared to non-narcissists [17,102].

“Selfie-taking may operate as a modern type of body checking where people compare their image with sociocultural standards” [50] (p. 78). Study 3 further attempts to explore the relationship between physical appearance comparison and selfie/groupfie posting on Instagram, drawing from social comparison theory [97] and evolutionary psychology. According to social comparison theory [97], people evaluate their own physical appearance by comparison with the sociocultural ideals presented in the media. Social comparison is even more pertinent to social media than it is to traditional media because of the speed, ease, and technological affordance of making frequent, multiple, and rapid comparisons in real time [48,52]. Driven by evolutionary psychology of narcissism [82,95] discussed above, it can be hypothesized that selfie-posting is a self-promotion strategy deployed by narcissists in the process of physical appearance comparison and intrasexual competition for mates in social media environments (H5). People use social comparison information to form cost-effective strategies that guide intrasexual competitive behaviors [96,100]. The relationships between selfie behavior and body dissatisfaction are bidirectional, with selfie-sharing both preceding and resulting from appearance dissatisfaction [49]. Ultimately, this study proposes the frequency of taking selfies/groupfies (H6a) and quantitative indexes of Instagram selfie/groupfie posts (H6b) as the final endogenous outcome variables, resulting from the multifaceted human desires cultivated via Instagram as well as attitudinal factors [15]:

**H5:**  *Instagram users’ physical appearance comparison and intrasexual competition for mates are positive predictors of (a) attitude toward selfies/groupfies and (b) intention to post selfies/groupfies on Instagram*.

**H6:**  *Instagram users’ intention to post selfies/groupfies is a positive predictor of (a) frequency of posting selfies/groupfies and (b) the number of selfie/groupfie posts on Instagram*.

**Figure 3:**
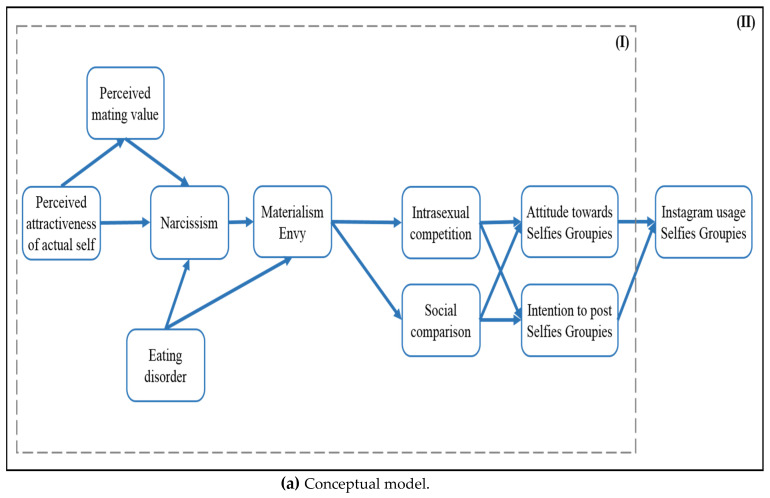

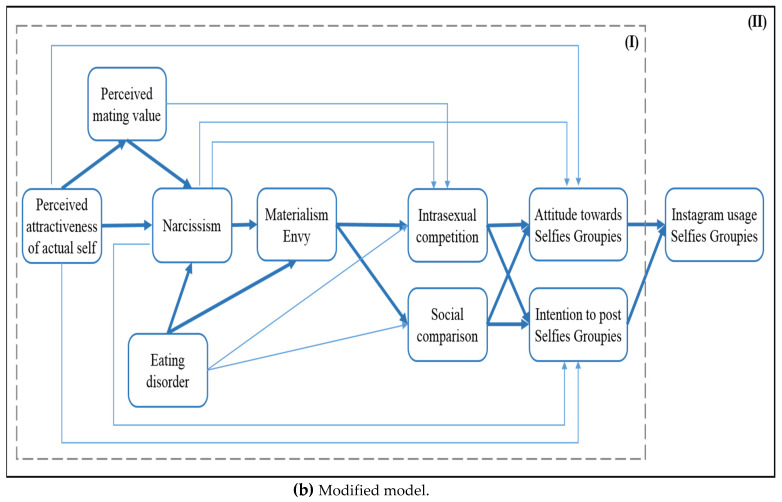
Study 3: Conceptual model (**a**) and modified model (**b**).

An integrative model at the global level graphically visualizing local-level individual hypotheses that propose the theorized relationships among the multifaceted human desires manifested and fulfilled via Instagram is presented in Figure 3a,b.

The conceptual model (Figure 3a with only thick lines) was modified to (Figure 3b with additional thin lines) to establish a well-fitting structural equation model. 

## 3. Materials and Methods

### 3.1. Study 1

#### 3.1.1. Participants and Data Collection

Participants (*N* = 108; 61 males and 47 females; *Mean* _Age_ = 32.84, *SD* _Age_ = 10.00) were recruited from MTurk for cross-sectional survey data collection. MTurk is a crowdsourcing website that recruits crowd workers to perform on-demand tasks such as research surveys. After submitting an informed consent form, participants were asked to fill out an online survey prepared on the Qualtrics platform.

#### 3.1.2. Measures

Exogenous variables include appearance-related self-confidence [103], and self-discrepancy [37] with regard to physical attractiveness [104]. Self-discrepancy was operationalized as the quantitative discrepancy between the ideal self and actual self [37] and therefore was measured by calculating the difference between participants’ ideal self and actual self with respect to physical attractiveness [104]. Materialism was measured with two operationalizations: Richins’ [105] three dimensions (success, acquisition, and happiness) and Belk’s [29] three dimensions (possession, nongenerosity, and envy). The outcome variable was Instagram usage intensity. Both the objective dimension (i.e., quantitative usage frequency) and the subjective dimension (i.e., perceived usage intensity) of Instagram usage were examined. 

Table 1 summarizes the number of items for each measure, the results of reliability testing (Cronbach’s alpha), and an example item for each variable measured in Study 1. Table 2 presents descriptive statistics. 

### 3.2. Study 2

#### 3.2.1. Participants and Data Collection

Participants (*N* = 140; 89 males and 51 females; *M*_Age_ = 33.27, *SD* _Age_ = 8.68) were recruited from MTurk for cross-sectional survey data collection. After submitting an informed consent form, participants were asked to fill out an online survey.

#### 3.2.2. Measures

Exogenous variables include one’s own weight status perception [59] and self-esteem [106]. Eating disorder measures consist of three dimensions: perfectionism [107], anorexia [108], and bulimia nervosa [108]. Two types of envy were measured: malicious envy and benign envy [109]. Instagram usage was measured with two operationalizations: objective and quantitative measure of usage frequency and subjective perception of usage intensity [110]. 

Table 3 summarizes the number of items for each measure, the results of reliability testing (Cronbach’s alpha), and an example item for each variable measured in Study 2. Table 4 presents descriptive statistics.

### 3.3. Study 3

#### 3.3.1. Participants and Data Collection

Participants (*N* = 557) were sampled from Amazon MTurk (age ranged from 18 to 76, *M* _Age_ = 33.18, *SD*_Age_ = 10.17; ethnic composition: 52.42% White, 31.78% Asian, 5.75% African American, 5.75% American Indian or Alaska Native, 0.54% Native Hawaiian or other Pacific Islander, 3.77% Other; gender composition: 331 males (59.43%) and 226 females (40.57%)) for cross-sectional survey data collection. For Model 2, data from 375 participants, who entered valid responses to the numbers of Instagram selfie posts and groupfie posts, were analyzed (*N* = 375, age ranged from 20 to 70, *Mean*_Age_ = 32.08, *Median* _Age_ = 30, *SD* _Age_ = 8.60; ethnic composition: 53.60% White, 30.13% Asian, 5.07% African American, 6.67% American Indian or Alaska Native, 0.53% Native Hawaiian or other Pacific Islander, 4% Other; gender composition: 221 males (58.93%) and 154 females (41.07%)). Participants completed an informed consent form and filled out an online questionnaire prepared on the Qualtrics platform. 

#### 3.3.2. Measures

Self-perceived physical attractiveness (SPPA) was measured with attractiveness scales [104]. Perfectionism [PFT], anorexia [AN], and bulimia nervosa [BN] were measured with Hill et al.’s [107] perfectionism scale and Friborg, Clausen, and Rosenvinge’s [108] Eating Disorder Inventory (EDI−3), using 7-point Likert scales ranging from “strongly disagree” [1] to “strongly agree” [7]. Narcissistic grandiosity was measured with the Narcissistic Grandiosity Scale (NGS) [111], using 7-point semantic differential scales. Envy was measured with the envy dimension of materialism scales [29]. Perceived mate value (PMV) was measured with the Self-Perceived Mating Success Scale (SPMSS) [112], using 7-point Likert scales. Intrasexual competition for mates (ISC) was measured with intrasexual competition scales [113]. Social comparison was measured with the Social Comparison Orientation Scale (SCOS) [114], using 7-point Likert scales. Physical appearance comparison was measured with the Physical Appearance Comparison Scale (PACS) [115], using 7-point Likert scales. Attitude toward selfies/groupfies was measured with attitude scales proposed in the theory of planned behavior [15,116]. Intention to post selfies/groupfies was measured with intention scales proposed in the theory of planned behavior [116]. The numbers of selfie posts and groupfie posts on Instagram were measured by asking participants to enter the exact number of their Instagram posts. 

The number of items for each variable and the results of reliability testing (Cronbach’s alpha) are shown in Table 5. The means (*M*) and standard deviations (*SD*) and the results of correlation analyses are shown in Table 6 and Table 7. 

## 4. Results

### 4.1. Study 1

**Figure 4:**
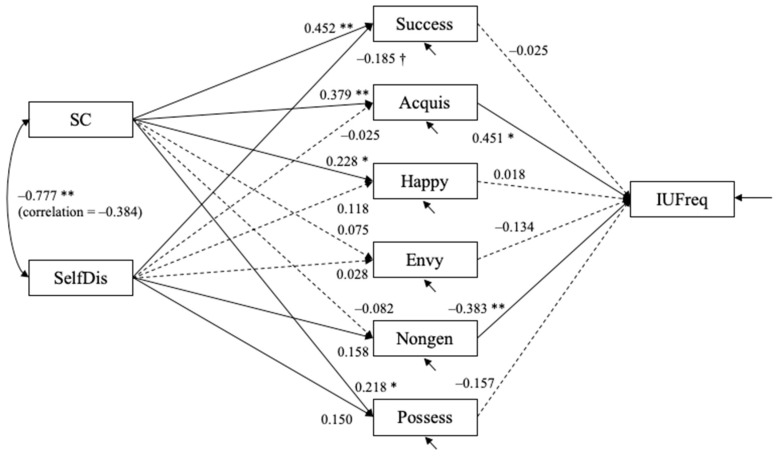
Study 1: Estimated structural equation model with objective measure of Instagram usage (*N* = 108).

**Figure 5:**
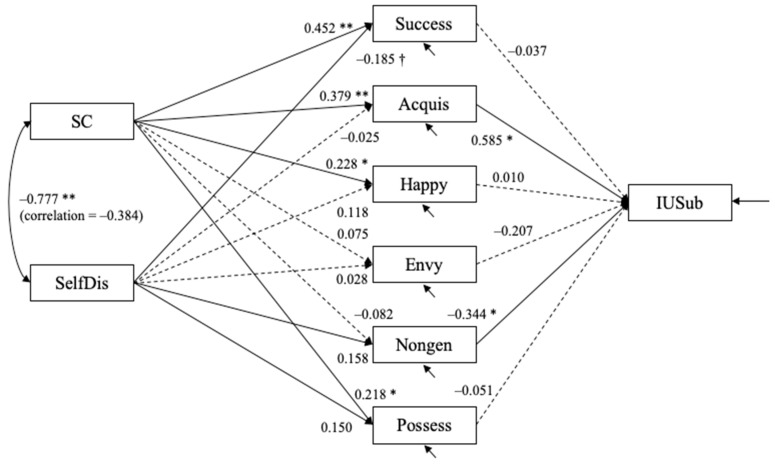
Study 1: Estimated structural equation model with subjective measure of Instagram usage (N = 108).

Following the way prominent scholars utilize structural equation modeling techniques to analyze survey data as well as adhering to the established scientific methods of proposing individual hypotheses at the local level and then testing the integrative models at the global level, data were analyzed using structural equation modeling methods. Structural equation models were estimated using Mplus 8. The hypothesized models fit well. For the model with the objective measure of Instagram usage (IUFreq), χ2(7) = 10.085, *p* = 0.184, CFI = 0.988, RMSEA = 0.064 with 90% confidence interval = (0.000, 0.144), and SRMR = 0.051. For the model with the subjective measure of Instagram usage (IUSub), χ^2^(7) = 10.463, *p* = 0.164, CFI = 0.986, RMSEA = 0.068 with 90% confidence interval = (0.000, 0.147), and SRMR = 0.051. Figure 4 and Figure 5 show the estimated structural equation models with the objective and subjective measures of usage, respectively. In both models, self-confidence positively predicted success (bˆ = 0.452, SE = 0.108, *p* < 0.001), acquisition (bˆ = 0.379, SE = 0.093, *p* < 0.001), happiness (bˆ = 0.228, SE = 0.099, *p* = 0.021), and possession (bˆ = 0.218, SE = 0.090, *p* = 0.015) dimensions of materialism. Self-discrepancy was a statistically significant predictor of only the success (bˆ = −0.185, SE = 0.105) dimension of materialism.

The objective measure of usage was positively predicted by acquisition (bˆ = 0.451, SE = 0.213, *p* = 0.034) and negatively predicted by nongenerosity (bˆ = −0.383, SE = 0.146, *p* = 0.009). The subjective measure of usage was also positively predicted by acquisition (bˆ = 0.585, SE = 0.207, *p* = 0.005) and negatively predicted by nongenerosity (bˆ = −0.344, SE = 0.142, *p* = 0.016).

Unstandardized estimates are shown. Dashed line depicts path coefficients for which *p* > 0.10; † *p* < 0.08; * *p* < 0.05; ** *p* < 0.01. The residuals for five materialism variables (except for materialism possession) are allowed to covary with one another (not shown in the figure).

Unstandardized estimates are shown. Dashed line depicts path coefficients for which *p* > 0.10; † *p* < 0.08; * *p* < 0.05; ** *p* < 0.01. The residuals for five materialism variables (except for materialism possession) are allowed to covary with one another (not shown in the figure). 

### 4.2. Study 2

**Figure 6:**
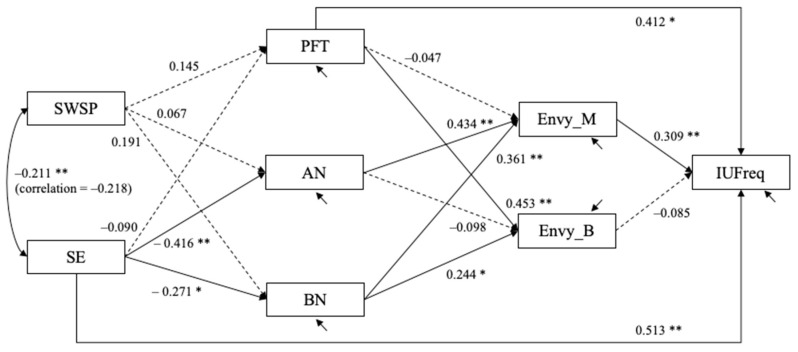
Study 2: Estimated structural equation model with objective measure of Instagram usage (N = 140).

**Figure 7:**
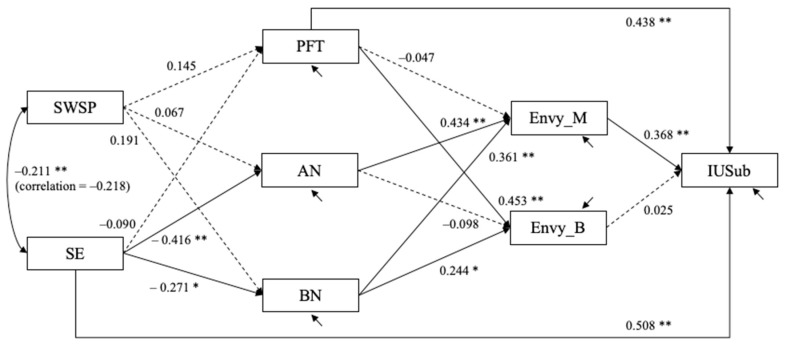
Study 2: Estimated structural equation model with subjective measure of Instagram usage (N = 140).

Structural equation models were estimated to test the hypothesized model using Mplus 8. The initial models specified based on the conceptual model resulted in the following model fit statistics. For the model with the objective measure of Instagram usage (IUFreq), χ^2^(9) = 31.616, *p* < 0.001, CFI = 0.932, RMSEA = 0.134 with 90% confidence interval = (0.085, 0.186), and SRMR = 0.062. For the model with the subjective perception of Instagram usage (IUSub), χ^2^(9) = 36.277, *p* < 0.001, CFI = 0.921, RMSEA = 0.147 with 90% confidence interval = (.099, 0.199), and SRMR = 0.065. Both models were modified by allowing the direct paths from self-esteem to Instagram usage and from perfectionism to Instagram usage freely estimated. Figure 6 and Figure 7 show the estimated structural equation models with the objective and subjective measures of usage, respectively. The models fit the data well. For the model with the objective measure of usage, χ^2^(7) = 11.879, *p* = 0.105, CFI = 0.985, RMSEA = 0.071 with 90% confidence interval = (0.000, 0.138), and SRMR = 0.032. For the model with the subjective perception of usage, χ^2^(7) = 10.746, *p* = 0.15, CFI = 0.989, RMSEA = 0.062 with 90% confidence interval = (0.000, 0.131), and SRMR = 0.030. In both models, self-esteem was negatively related to anorexia (unstandardized estimate bˆ = −0.416, standard error (SE) = 0.089, *p* < 0.001) and bulimia nervosa (bˆ = −0.271, SE = 0.107, *p* = 0.011). Anorexia (bˆ = 0.434, SE = 0.115, *p* < 0.001) and bulimia nervosa (bˆ = 361, SE = 0.095, *p* = 0.011) were positively related to malicious envy, which then was positively related to the Instagram usage (bˆ = 0.309, SE = 102, *p* = 0.002 for IUFreq; bˆ = 0.368, SE = 089, *p* < 0.001 for IUSub). 

Unstandardized estimates are shown. Dashed line depicts path coefficients for which *p* > 0.10; † *p* < 0.08; * *p* < 0.05; ** *p* < 0.01. The residuals for three eating disorder variables (PFT, AN, BN) are allowed to covary with one another (not shown in the figure); the residuals for malicious envy and benign envy are allowed to covary with each other (not shown in the figure). 

Unstandardized estimates are shown. Dashed line depicts path coefficients for which *p* > 0.10; † *p* < 0.08; * *p* < 0.05; ** *p* < 0.01. The residuals for three eating disorder variables (PFT, AN, BN) are allowed to covary with one another (not shown in the figure); the residuals for malicious envy and benign envy are allowed to covary with each other (not shown in the figure). 

### 4.3. Study 3

Structural equation models were estimated using Mplus 8. The analysis was conducted in two steps. First, the conceptual model (I) for the psychological variables was evaluated (N = 557). Once a well-fitting model was established in (I), the Instagram usage variables were introduced as shown in the conceptual model (II). In the second step, two models were estimated: one with frequency of posting selfies and groupfies as measures of Instagram usage (Model II−1, N = 557), and the other with numbers of selfies and groupfies as measures of Instagram usage (Model II−2, N = 375 who had uploaded selfies and groupfies to their Instagram accounts and provided valid responses to the numbers of selfies and groupfies). In Model II−2, the numbers of selfies and groupfies were treated as count variables with negative binomial distribution because these responses were extremely positively skewed.

**Figure 8:**
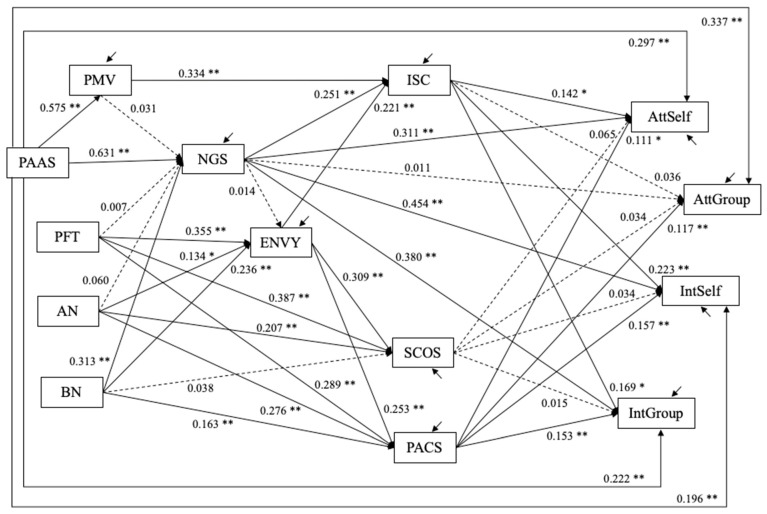
Study 3: Estimated structural equation model (Model I, N = 557).

Model I (N = 557). The initial model specified based on the conceptual model resulted in the following model fit statistics: χ^2^(51) = 784.403, *p* < 0.001, CFI = 0.817, RMSEA = 0.161 with 90% confidence interval = (0.151, 0.171), and SRMR = 0.180. The model was modified by removing constraints on the following path coefficients: from perceived mating value and narcissism to intrasexual competition (PMV → ISC, NGS → ISC); from eating disorder to intrasexual competition (PFT → ISC, AN → ISC, BN → ISC); from eating disorder to social comparison (PFT → SCOS, AN → SCOS, BN → SCOS, PFT → PACS, AN → PACS, BN → PACS); from narcissism and perceived attractiveness of actual self to attitude toward and intention to post selfies and groupfies (NGS → AttSelf, NGS → AttGroup, NGS → IntSelf, NGS → IntGroup, PAAS → AttSelf, PAAS → AttGroup, PAAS → IntSelf, PAAS → IntGroup). The modified model fit well: χ^2^(35) = 135.434, *p* < 0.001, CFI = 0.975, RMSEA = 0.072 with 90% confidence interval = (0.059, 0.085), and SRMR = 0.051. Compared to the initial model, the chi-squared statistic was reduced by 648.969 for the difference in degrees of freedom 16 (*p* < 10^−127^). The estimates are shown in Figure 8. 

**Figure 9:**
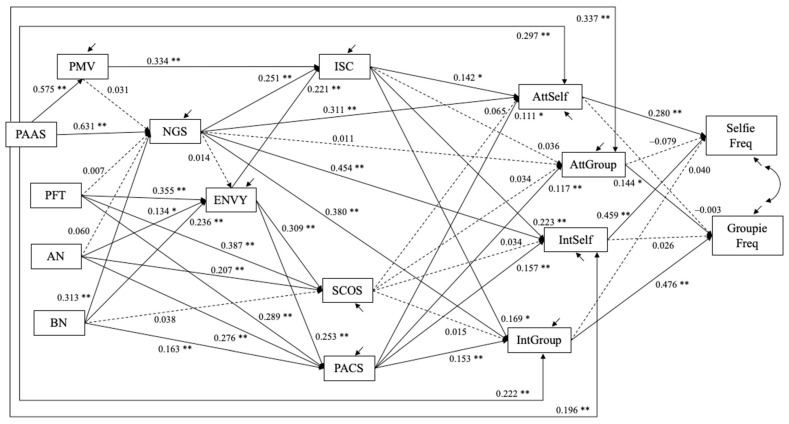
Study 3: Estimated structural equation model (Model II-1, N = 557).

Model II−1 (N = 557). The frequency of posting selfies and groupfies (1–7 scale) were used as measures of Instagram usage. The model fit statistics were: χ^2^(55) = 220.804, *p* < 0.001, CFI = 0.966, RMSEA = 0.074 with 90% confidence interval = (0.064, 0.084), and SRMR = 0.060. The estimated model is shown in Figure 9. The frequency of posting selfies was positively predicted by attitude (bˆ = 0.280, SE = 0.057, *p* < 0.001) toward and intention (bˆ = 0.459, SE = 0.048, *p* < 0.001) to post selfies, but not by attitude toward (bˆ = −0.079, SE = 0.054, *p* = 0.143) and intention (bˆ = 0.040, SE = 0.042, *p* = 0.346) to post groupfies. Likewise, the frequency of posting groupfies was positively predicted by groupfie attitude (bˆ = 0.144, SE = 0.061, *p* = 0.018) and intention (bˆ = 0.476, SE = 0.048, *p* < 0.001), but not selfie attitude (bˆ = −0.003, SE = 0.064, *p* = 0.962) and intention (bˆ = 0.026, SE = 0.054, *p* = 0.627). 

Unstandardized estimates are shown. Dashed line depicts path coefficients for which *p* > 0.10; † *p* < 0.08; * *p* < 0.05; ** *p* < 0.01. The following covariances are allowed in the model but not shown in the figure: perceived mating value (PMV) and three eating disorder variables (PFT, AN, BN) are allowed to covary with one another; the residuals for intrasexual competition (ISC) and social comparison (SCOS, PACS) are allowed to covary with one another; the residuals for attitudes toward and intention to take selfies and groupfies (AttSelf, AttGroup, IntSelf, IntGroup) are allowed to covary with one another.

Unstandardized estimates are shown. Dashed line depicts path coefficients for which *p* > 0.10; † *p* < 0.08; * *p* < 0.05; ** *p* < 0.01. The following covariances are allowed in the model but not shown in the figure: perceived mating value (PMV) and three eating disorder variables (PFT, AN, BN) are allowed to covary with one another; the residuals for intrasexual competition (ISC) and social comparison (SCOS, PACS) are allowed to covary with one another; the residuals for attitudes toward and intention to take selfies and groupfies (AttSelf, AttGroup, IntSelf, IntGroup) are allowed to covary with one another.

**Model II-2 (N = 375).** The number of selfies ranged from 0 to 654, the 25th percentile = 2, median = 8, the 75-th percentile = 29. The number of groupfies ranged from 0 to 454, the 25th percentile = 1, median = 6, the 75th percentile = 22. The distribution of these count (i.e., how many) responses was positively skewed with long upper tails. In Model II-2, these outcomes were treated as count variables with negative binominal distribution to take into account the over dispersion. 

Before estimating Model II-2, Model I was estimated again with the subset of data from 375 participants who had uploaded selfies and groupfies to their Instagram accounts and provided valid responses to the numbers of selfies and groupfies. The model fit the data well: χ^2^(35) = 94.957, df = 140, *p* < 0.001; CFI = 0.977; RMSEA = 0.068 with 90% confidence interval = (.051, 0.084); SRMR = 0.046. 

**Figure 10:**
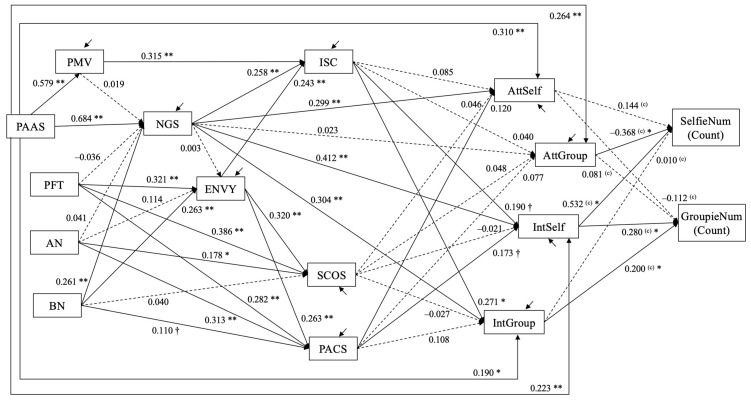
Study 3: Estimated structural equation model (Model II-2, *N* = 357).

Then, Model II-2 was tested with the numbers of selfies and groupfies. The estimated model is shown in Figure 10. The estimates of the coefficients on the count variables indicate the difference in the log of count response. For the number of selfies (SelfieNum), the log of the number was larger by 0.532 (SE = 0.093, *p* < 0.001), or the incident rate of posting a selfie increased by e ^0.532^ = 1.702 times per 1 higher score in intention to post selfies. Given that the standard deviation (SD) of intention to post selfies was 2.045, the incident rate of posting a selfie increased by *e* ^0.532*2.045^ = 2.968 times per 1 SD difference in intention to post selfies. For 1 higher score in attitude toward groupfies, the log of the number of selfies was smaller by 0.368 (SE = 0.172, *p* = 0.033), which means that the incident rate of posting selfies decreased by *e* ^−0.368^ = 0.692 times per 1 score difference or the incident rate decreased by *e* ^−0.368*1.353^ = 0.608 times per 1SD (1.353) difference in attitude toward groupfies.

For the number of groupfies (GroupfieNum), the log of the number was larger by 0.280 (SE = 0.111, *p* = 0.012), which means that the incident rate of posting groupfies increased by *e* ^0.280^ = 1.323 times per 1 score difference or *e* ^0.280*2.045^ = 1.773 times per 1SD (2.045) difference in intention to post groupfies. For 1-higher scores in intention to take groupfies, the log of the number of groupfies was larger by 0.200 (SE = 0.090, *p* = 0.025), which means that the incident rate of posting groupfies increased by *e* ^0.200^ = 1.221 times per 1 score difference or *e* ^0.200*1.902^ = 1.463 times per 1SD (1.902) difference in intention to take groupfies.

Unstandardized estimates are shown. Dashed line depicts path coefficients for which *p* > 0.10; † *p* < 0.08; * *p* < 0.05; ** *p* < 0.01. The variables SelfieNum and GroupfieNum were treated as count variables with negative binomial distribution. The coefficients on the count variables (marked with superscript “(C)”) indicate the difference in the log of the count response. The following covariances are allowed in the model but not shown in the figure: perceived mating value (PMV) and three eating disorder variables (PFT, AN, BN) are allowed to covary with one another; the residuals for intrasexual competition (ISC) and social comparison (SCOS, PACS) are allowed to covary with one another; the residuals for attitudes toward and intention to take selfies and groupfies (AttSelf, AttGroup, IntSelf, IntGroup) are allowed to covary with one another. 

## 5. Discussion

### 5.1. Significance and Key Findings

The current findings highlight the significance of examining a variety of predictors of Instagram usage and selfie/groupfie behaviors drawing from an evolutionary psychological perspective. Evolutionary approaches have great heuristic value, which is defined as “the potential to stimulate or encourage further thinking” [117], in guiding behavioral scientists to produce a wealth of discoveries about hardwired human cognition, emotion, perception, and behavior [118]. Thus, the current research is an attempt to provide an evolutionary explanation of the Instagram culture, which has been examined from critical perspectives in the consumption markets and culture literature [12]. 

Much of the current findings are novel and have never been reported in the previous empirical studies. Study 1 demonstrates relationships among self-confidence, self-discrepancy, materialism, and Instagram usage. Appearance-related self-confidence is a positive predictor of success, acquisition, and happiness dimensions of materialism, whereas appearance-related self-discrepancy is a negative predictor of the success dimension of materialism, which has not been reported and differentiated in the prior literature. The acquisition dimension of materialism positively predicts Instagram usage intensity, whereas the nongenerosity dimension of materialism negatively predicts Instagram usage.

Study 2 demonstrates associations among people’s weight status perception, self-esteem, eating disorder, envy, and Instagram usage intensity. Self-esteem is a negative predictor of multiple dimensions of eating disorder (perfectionism, anorexia, and bulimia nervosa), which is consistent with the previous literature [119]. Eating disorders, in turn, predict malicious envy and benign envy, which also reconfirms the previous literature [21]. The most important novel finding from Study 2 is that malicious envy is a positive predictor of both quantitative Instagram usage frequency and subjective perception of Instagram usage intensity. 

Study 3 sought to explore the link between body image perception and narcissistic selfie/groupfie-posting behavior. The results of a series of structural equation modeling analyses support each individual hypothesis at the local level as well as demonstrate an excellent model fit at the global model. At the local level, self-perceived physical attractiveness predicts narcissistic grandiosity and self-perceived mating value. Eating disorder predicts narcissism, envy, social comparison, and physical appearance comparison. Narcissistic grandiosity predicts intrasexual competition for mates. Materialistic envy predicts social comparison, physical appearance comparison, and intrasexual competition for mates. Ultimately, narcissistic grandiosity, intrasexual competition for mates, and physical appearance comparison predict intention to post selfies/groupfies, which in turn predicts quantitative indexes of actual selfie/groupfie post counts on Instagram. Overall, the global models fit the data well. Building upon the previous findings about the selfie-and-groupfie culture from the Freudian psychological perspective [15], the current study proposes more sophisticated models as well as provides richer empirical data on a wide variety of dispositional, psychological, and attitudinal predictors of Instagram usage behaviors and selfie-and-groupfie usage behaviors.

### 5.2. Theoretical Contributions and Practical Implications

To answer the “why” questions as a quest for the fundamental causes of Instagram usage behaviors, the current study draws from the theoretical framework of evolutionary psychology. Drawing from a unique evolutionary psychological perspective, this research presents findings from three sets of empirical data about basic human needs and desires that motivate various dimensions of Instagram usage behaviors. Study 1 makes theoretical contributions to consumer psychology of materialism in Web 2.0 environments. Despite the abundant literature on the impact of social media usage on body image [6,53,54], no prior research discovered processing mechanisms through which Instagram users’ body image-related self-confidence and self-discrepancy predict multiple dimensions of materialism. Study 1 adds new empirical findings to the literature as well as provides a fresh perspective on the association among Instagram users’ body image perception, their materialistic values, and Instagram usage intensity. Study 2 makes theoretical contributions to the extant literature on body image, eating disorder, and envy [8,19,22] by examining the underlying mechanisms whereby body image perception and self-esteem jointly influence multiple dimensions of eating disorders. It also adds interesting and novel findings about the association between malicious envy and Instagram usage intensity. Study 3 offers new insights into the dynamic relationships among subjective body image perception, eating disorder, mating value, narcissism, envy, social comparison, physical appearance comparison, and selfie/groupfie posting. The inclusion of perceived mate value and intrasexual competition for mates in an integrative model of body image, narcissism, and Instagram usage is novel and marks a more sophisticated refinement of the extant model [15]. This novel approach to the evolutionary psychological perspective on narcissism adds to our theoretical understanding of the fundamental mechanism that explains “*why*” narcissism is associated with body image, physical appearance comparison, mating value, intrasexual competition for mates, and ultimately with selfie/groupfie-posting behavior on Instagram. The integrative model theorized and empirically tested in the current study not only demonstrates the manifestation of a variety of fundamental and evolutionarily hardwired human desires as motivators of Instagram usage [120] but also implies that narcissistic selfie/groupfie posting behavior can be interpreted as an evolutionarily adaptive self-promotion strategy in intrasexual competition for mates in excessively appearance-focused social media environments [121].

In addition to these theoretical contributions to the literature, this study also has practical implications for marketing communication and health communication. With regard to brand management and marketing communication, results from Study 1 indicate that materialistic consumers who score high on acquisition dimension tend to use Instagram more than nonmaterialistic consumers, which implicates that those managers of luxury brands and social media marketers can strategically target materialistic consumers by exposing them to photos of luxury brands’ products. With regard to health communication about eating disorders, healthy dieting, and the obesity epidemic, results from Study 2 indicate that people with high levels of anorexia and bulimia nervosa tend to score high on malicious envy, which, in turn, increases Instagram usage intensity. Public health professionals may specifically target frequent Instagram users with eating disorders and malicious envy by embedding visual images (food photos and foodies’ body images) for disseminating relevant health communication messages about healthy diet and physical exercise. Study 3 offers practical implications for online dating apps/SNSs as well as managerial implications for the online dating industry and marketing communication in general. Marketing research shows that social media usage elicits narcissism and envy, which in turn increases consumers’ desire for self-promotion and propensity to engage in conspicuous consumption [122]. The current findings about the associations among envy, social comparison, and narcissistic self-promotion in the form of selfie/groupfie posting, and their combined influence on social media usage (i.e., measured by quantitative indicators of selfie/groupfie posting frequency and actual number of Instagram selfie/groupfie posts) are consistent with the emerging stream of research on the potential of social media for marketing communication [20]. With specific regard to managerial implications for online dating apps/SNSs and the online dating industry, the current findings suggest that consumers’ physical attraction, intrasexual competition for mates, perceived mating value, and physical appearance comparison are integral factors that motivate them to sign up for online dating apps/SNSs and upload attractive profile photos. Social media marketers and brand managers of online dating apps/SNSs can substantially profit from the solid understanding of these motivational factors in attracting paying subscribers, increasing profile photo uploads, and ultimately boosting website traffic and revenue.

### 5.3. Limitations and Suggestions for Future Research

This research is not without limitations. First, given the relevance of Instagram as a visual image-based platform for body image perception, this survey particularly focused on people’s Instagram usage and selfies/groupfies posted on Instagram. Follow-up studies need to measure participants’ use of other social media platforms (TikTok, Pinterest, Facebook, Snapchat, Tumblr, etc.) and appearance-focused online dating apps (Tinder, Bumble, Facebook Dating, etc.) not only to increase external validity but also to compare among various social media platforms/apps with regard to body image perception and selfie/groupfie posting frequency. Second, the cross-sectional nature of this survey study limits the conclusions that can be drawn regarding causality and directionality of results. Building upon the current study’s theoretical foundations and empirical findings, future research can experimentally test the causal relationship between narcissism and body-related visual image posting on social media. Future studies can prime various aspects of participants’ narcissism and measure their instant behavioral reaction, operationalized by actual body-related selfie/groupfie taking/posting behaviors in an experimental setting, in real-time. For example, future research would benefit from creative methods such as experimentally manipulating body-related visuals and priming specific dimensions (exhibitionism/vanity, authority/leadership, exploitativeness/entitlement) of narcissism [123]. Third, all the data were based on self-report survey questionnaire. This line of future research needs to develop more objective methods of measuring people’s body image perception, eating disorder, narcissism, and envy to provide more valid behavioral and physiological data. Additionally, the reliability (Cronbach’s alpha) score of the nongenerosity dimension of materialism is too low. This issue, unfortunately, cannot be fixed with the current datasets. Follow-up research needs to consider using more reliable measuring items and recompute the proposed structural equation models. Lastly, although this study measured multiple operationalizations of Instagram usage (quantitative usage frequency, subjective perception of usage intensity, selfie/groupfie post counts), the surveys did not measure other quantitative indexes such as the numbers of followers, followings, and total posts/comments/likes. Inclusion of these quantitative metrics in the follow-up studies will enable researchers to conduct more methodologically sophisticated surveys and collect data on various dimensions (e.g., popularity, social influence, and interactivity) of quantitative indexes of Instagram usage intensity.

## 6. Conclusions

Despite several limitations, this study provides theorists and practitioners with rich empirical data on the dynamic association among body image, eating disorder, materialism, envy, narcissism, Instagram usage frequency, and selfie/groupfie posting in excessively appearance-focused social media environments. As a result, this timely study addresses the pressing issues of societal concerns about mental health issues of Instagram users. From the unique standpoint of evolutionary theory, it also provides keen insights into people’s motivations for using social media and posting selfies in relation to fundamental human desires. In conclusion, this study has the potential to stimulate provocative discourses about a wide range of impact that social media contents have on people (“how and what”) as well as fundamental and multifarious motivations for using social media (“why”).

## Figures and Tables

**Table 1:** Study 1: Number of items, Cronbach’s alpha, and an example item for each measure.

Measure	Number of Items	Cronbach’s Alpha	Example Item
Self-confidence (SC)	4	0.912	“I feel confident about myself.”
Ideal self (PAIS)	5	0.949	“not sexy”–“sexy”
Actual self (PAAS)	5	0.908	“not sexy”–“sexy”
Self-discrepancy (SelfDis) ^a^			PAIS–PAAS
Materialism success (Success)	3	0.884	“I admire people who own expensive homes, cars, and clothes.”
Materialism acquisition (Acquis)	3	0.774	“Buying things gives me a lot of pleasure.”
Materialism happiness (Happy)	3	0.842	“I’d be happier if I could afford to buy more things.”
Materialism possession (Possess)	3	0.614	“I never discard old pictures or snapshots.”
Materialism nongenerosity (Nongen)	3	0.595	“I don’t like to lend things, even to good friends.”
Materialism envy (Envy)	3	0.746	“When friends have things I cannot afford, it bothers me.”
Quantitative measure of Instagram usage (IUFreq)	1		How often do you use Instagram?“never”–“always”
Subjective perception of Instagram usage (IUSub)	6	0.955	“I feel out of touch when I have not logged onto Instagram for a while.”

^a^ SelfDis = PAIS–PAAS.

**Table 2:** Study 1: Means, standard deviations (SDs), and correlations (Pearson’s parametric correlation) of variables (*N* = 108).

			Correlation								
Variable	*Mean*	*SD*	1	2	3	4	5	6	7	8	9
1. Self-confidence (SC)	4.71	1.40									
2. Self-discrepancy (SelfDis)^a^	0.77	1.46	−0.38 **								
3. Materialism success (Success)	4.10	1.65	0.45 **	−0.31 **							
4. Materialism acquisition (Acquis)	4.35	1.37	0.40 **	−0.18 *	0.75 **						
5. Materialism happiness (Happy)	5.16	1.37	0.19 *	0.04	0.40 **	0.46 **					
6. Materialism possession (Possess)	5.27	1.25	0.18 *	0.08	0.13 *	0.16 *	0.27 **				
7. Materialism nongenerosity (Nongen)	3.98	1.33	−0.15 *	0.21 **	0.16 *	0.18 *	0.30 **	0.12 *			
8. Materialism envy (Envy)	3.88	1.58	0.04	−0.03	0.44 **	0.44 **	0.39 **	0.06	0.40 **		
9. Quantitative measure of Instagram usage (IUFreq)	3.70	1.94	0.23 **	−0.24 **	0.13 *	0.19 *	−0.01	−0.10 *	−0.25 **	0.04	
10. Subjective perception of Instagram usage (IUSub)	3.60	1.92	0.29 **	−0.23 **	0.18 *	0.26 **	0.03	−0.02	−0.23 **	0.08	0.86 **

** Correlation is significant at the 0.01 level (2-tailed). * Correlation is significant at the 0.05 level (2-tailed).

**Table 3:** Study 2: Number of items, Cronbach’s alpha, and an example item for each measure.

Measure	Number of Items	Cronbach’s Alpha	Example Item
Self-weight status perception (SWSP)	1		“I perceived my body image as….” underweight [1]–average [2]–overweight [3]–obese [4].
Self-esteem (SE)	5	0.938	“I feel that I have a number of good qualities.”
Perfectionism (PFT)	8	0.726	“I get upset when other people do not maintain the same standards I do.”
Anorexia (AN)	5	0.853	“I feel bloated after eating a normal meal.”
Bulimia nervosa (BN)	4	0.894	“I have gone on eating binges where I felt that I could not stop.”
Malicious envy (Envy_M)	5	0.947	“If other people have something that I want for myself, I wish to take it away from them.”
Benign envy (Envy_B)	5	0.915	“I strive to reach other people’s superior achievements.”
Quantitative measure of Instagram usage (IUFreq)	1		How often do you use Instagram?“never”–“always”
Subjective perception of Instagram usage (IUSub)	6	0.956	“I feel out of touch when I have not logged onto Instagram for a while”

**Table 4:** Study 2: Means, standard deviations (SDs), and correlations (Pearson’s parametric correlation) of variables (*n* = 140).

			Correlation							
Variable	*Mean*	*SD*	1	2	3	4	5	6	7	8
1. Self-weight status perception (SWSP)	3.36	0.75								
2. Self-esteem (SE)	5.26	1.30	−0.22 **							
3. Perfectionism (PFT)	4.43	0.94	0.14 *	−0.15 *						
4. Anorexia (AN)	3.09	1.45	0.12 *	−0.38 **	0.49 **					
5. Bulimia nervosa (BN)	2.69	1.66	0.13 *	−0.23 **	0.39 **	0.78 **				
6. Malicious envy (Envy_M)	2.89	1.63	−0.03	−0.14 *	0.30 **	0.66 **	0.66 **			
7. Benign envy (Envy_B)	4.37	1.52	0.09	−0.01	0.34 **	0.25 **	0.30 **	0.31 **		
8. Quantitative measure of Instagram usage (IUFreq)	3.91	2.01	−0.10 *	0.27 **	0.20 *	0.14 *	0.25 **	0.24 **	0.08	
9. Subjective perception of Instagram usage (IUSub)	3.47	1.87	−0.01	0.28 **	0.27 **	0.20 *	0.31 **	0.35 **	19 *	0.86 **

** Correlation is significant at the 0.01 level (2-tailed). * Correlation is significant at the 0.05 level (2-tailed).

**Table 5:** Study 3: Number of items, Cronbach’s alpha, and an example item for each measure.

Measure	Number of Items	Cronbach’s Alpha	Example Item
Perceived mating value (PMV)	5	0.907	“I can have as many sexual partners as I choose.”
Perceived attractiveness of actual self (PAAS)	5	0.928	“attractive” “beautiful” “sexy”
Perfectionism (PFT)	5	0.669	“I spend a lot of time worrying about things I’ve done or things I need to do.”
Anorexia (AN)	4	0.854	“I exaggerate the importance of weight.”
Bulimia nervosa (BN)	3	0.898	“I eat moderately in front of others and stuff myself when they’re gone.”
Rosenthal Narcissistic Grandiosity Scale (NGS)	16	0.977	“omnipotent” “heroic” “powerful” “prestigious”
Materialism Envy (ENVY)	3	0.775	“When friends have things I cannot afford, it bothers me.”
Intrasexual competition (ISC)	4	0.747	“If a competitor thinks I am attractive, he/she will stay away from my romantic partner.”
Social comparison (SCOS)	3	0.889	“I always pay a lot of attention to how I do things compared with how others do things.”
Physical appearance comparison (PACS)	3	0.931	“I compare my physical appearance to the physical appearance of others.”
Attitude toward selfies (AttSelf)	5	0.948	“pleasant” “exciting” “beneficial”
Attitude toward groupfies (AttGroup)	5	0.947	“pleasant” “exciting” “beneficial”
Intention to post selfies (IntSelf)	2	0.956	“I will try to post a selfie on Instagram in the coming month.”
Intention to post groupfies (IntGroup)	2	0.954	“I will try to post a groupfie on Instagram in the coming month.”
Frequency of posting selfies (SelfieFreq)	1		“How often do you post selfies on Instagram?”
Frequency of posting groupfies (GroupfieFreq)	1		“How often do you post groupfies on Instagram?”
Number of selfies (SelfieNum) ^a^	1		“How many selfies have you uploaded on your Instagram account page?”
Number of groupfies (GroupfieNum) ^a^	1		“How many groupfies have you uploaded on your Instagram account page?”

^a^ Only the participants who had an Instagram account responded to these items (*N* = 375).

**Table 6:** Study 3: Means, standard deviations (SDs), and correlations (Pearson’s parametric correlation) of variables (*N* = 557).

Variable	*Mean*	*SD*	1	2	3	4	5	6	7	8	9	10	11	12	13	14	15
1. PMV	4.20	1.54															
2.PAAS	4.63	1.47	0.55 **														
3. PFT	4.48	1.11	0.13 *	0.10 *													
4. AN	3.41	1.62	0.13 *	0.06	0.49 **												
5. BN	2.79	1.81	0.13 *	0.13 *	0.33 **	0.73 **											
6. NGS	3.67	1.68	0.38 **	0.62 **	0.20 **	0.34 **	0.46 **										
7. ENVY	3.82	1.53	0.00	0.06	0.42 **	0.48 **	0.47 **	0.24 **									
8. ISC	4.06	1.34	0.55 **	0.50 **	0.31 **	0.33 **	0.37 **	0.55 **	0.33 **								
9. SCOS	4.17	1.67	0.19 *	0.22 **	0.51 **	0.51 **	0.42 **	0.32 **	0.51 **	0.46 **							
10. PACS	3.94	1.84	0.23 **	0.21 **	0.47 **	0.56 **	0.51 **	0.32 **	0.48 **	0.51 **	0.75 **						
11. AttSelf	4.56	1.72	0.31 **	0.53 **	0.25 **	0.26 **	0.31 **	0.58 **	0.24 **	0.49 **	0.36 **	0.37 **					
12. AttGroup	5.25	1.40	0.25 **	0.42 **	0.28 **	0.13 *	0.09	0.31 **	0.12 *	0.32 **	0.25 **	0.28 **	0.67 **				
13. IntSelf	3.73	2.08	0.32 **	0.47 **	0.20 **	0.29 **	0.37 **	0.58 **	0.26 **	0.50 **	0.34 **	0.38 **	0.80 **	0.52 **			
14. IntGroup	4.06	1.98	0.27 **	0.45 **	0.20 **	0.24 **	0.31 **	0.54 **	0.21 **	0.45 **	0.31 **	0.35 **	0.64 **	0.63 **	0.74 **		
15. SelfieFreq	3.47	1.82	0.30 **	0.45 **	0.19 *	0.35 **	0.44 **	0.59 **	0.28 **	0.47 **	0.34 **	0.38 **	0.67 **	0.41 **	0.74 **	0.56 **	
16.GroupfieFreq	3.39	1.75	0.26 **	0.36 **	0.22 **	0.22 **	0.31 **	0.42 **	0.18 *	0.39 **	0.24 **	0.28 **	0.44 **	0.47 **	0.48 **	0.63 **	0.60 **

** Correlation is significant at the 0.01 level (2-tailed). * Correlation is significant at the 0.05 level (2-tailed).

**Table 7:** Study 3: Means, standard deviations (SDs), and correlations (Pearson’s parametric correlation) of variables (*N* = 357).

Variable	*Mean*	*SD*	1	2	3	4	5	6	7	8	9	10	11	12	13
1. PMV	4.26	1.48													
2. PAAS	4.70	1.46	0.57 **												
3. PFT	4.53	1.12	0.14 *	0.09											
4. AN	3.43	1.62	0.10 *	0.00	0.47 **										
5. BN	2.82	1.80	0.06	0.09	0.32 **	0.71 **									
6. NGS	3.82	1.64	0.38 **	0.64 **	0.14 *	0.24 **	0.36 **								
7. ENVY	3.81	1.52	0.01	0.03	0.39 **	0.46 **	0.48 **	0.18 *							
8. ISC	4.18	1.31	0.53 **	0.51 **	0.31 **	0.29 **	0.31 **	0.53 **	0.34 **						
9. SCOS	4.25	1.66	0.16 *	0.16 *	0.50 **	0.48 **	0.40 **	0.23 **	0.50 **	0.46 **					
10. PACS	4.06	1.82	0.21 **	0.14 *	0.46 **	0.56 **	0.48 **	0.22 **	0.47 **	0.50 **	0.75 **				
11. AttSelf	4.71	1.67	0.29 **	0.52 **	0.24 **	0.19 *	0.23 **	0.54 **	0.21 **	0.45 **	0.29 **	0.30 **			
12. AttGroup	5.37	1.35	0.22 **	0.35 **	0.28 **	0.09	0.06	0.27 **	0.08	0.28 **	0.21 **	0.21 **	0.66 **		
13. IntSelf	4.00	2.04	0.29 **	0.45 **	0.17 *	0.19 *	0.27 **	0.53 **	0.20 *	0.45 **	0.26 **	0.30 **	0.80 **	0.51 **	
14. IntGroup	4.25	1.90	0.26 **	0.42 **	0.16 *	0.16 *	0.24 **	0.47 **	0.18 *	0.44 **	0.22 **	0.26 **	0.63 **	0.63 **	0.72 **

** Correlation is significant at the 0.01 level (2-tailed). * Correlation is significant at the 0.05 level (2-tailed).

## Data Availability

The data presented in this study will be made available to researchers upon submission of an approved ethics protocol from their academic or research institution (or equivalent) to the corresponding author.

## References

[1] Statista: Number of Monthly Active Instagram Users from January 2013 to June 2018 (In Millions). https://www.statista.com/statistics/253577/number-of-monthly-active-instagram-users/.

[2] Dean B. Instagram Demographic Statistics: How Many People use Instagram in 2021?. https://backlinko.com/instagram-users#instagram-stats-top-picks.

[3] Statista: Instagram- Statistics & Facts. https://www.statista.com/topics/1882/instagram/#dossierKeyfigures.

[4] Phua J., Jin S.A. (2011). Finding a home away from home: The use of SNSs by Asia-Pacific students in the United States for bridging and bonding social capital. Asian J. Commun..

[5] Brown Z., Tiggemann M. (2016). Attractive celebrity and peer images on Instagram: Effect on women’s mood and body image. Body Image.

[6] Fardouly J., Pinkus R.T., Vartanian L.R. (2017). The impact of appearance comparisons made through social media, traditional media, and in person in women’s everyday lives. Body Image.

[7] Kim J.W., Chock T.M. (2015). Body image 2.0: Associations between social grooming on Facebook and body image concerns. Comput. Hum. Behav..

[8] Holland G., Tiggemann M. (2016). A systematic review of the impact of the use of social networking sites on body image and disordered eating outcomes. Body Image.

[9] Wells G., Horwitz J., Seetharaman D. (2021). Facebook Knows Instagram is Toxic for Teen Girls, Company Documents Show. Wall Str. J..

[10] Perrigo B. Instagram Makes Teen Girls Hate Themselves. Is That a Bug or Feature?. TIME 2021..

[11] Seetharaman D. (2021). Senators Seek Answers from Facebook after WSJ Report on Instagram’s Impact on Young Users. Wall Str. J..

[12] Murray D.C. (2015). Notes to self: The visual culture of selfies in the age of social media. Consum. Mark. Cult..

[13] Murray D.C. (2020). Selfie consumerism in a narcissistic age. Consum. Mark. Cult..

[14] Dumas T.M., Maxwell-Smith M., Davis J.P., Giulietti P.A. (2017). Lying or longing for likes? Narcissism, peer belonging, loneliness and normative versus deceptive like-seeking on Instagram in emerging adulthood. Comput. Hum. Behav..

[15] Jin S.V., Ryu E. (2018). “The paradox of Narcissus and Echo in the Instagram Pond” in light of the selfie culture from Freudian evolutionary psychology: Self-loving and confident but lonely. J. Broadcast. Electron. Media..

[16] Tiggemann M., Hayden S., Brown Z., Veldhuis J. (2018). The effect of Instagram “likes” on women’s social comparison and body dissatisfaction. Body Image.

[17] Jin S.V., Muqaddam A. (2018). “Narcissism 2.0! Would narcissists follow fellow narcissists on Instagram?” the mediating effects of narcissists personality similarity and envy, and the moderating effects of popularity. Comput. Hum. Behav..

[18] Jin S.V., Muqaddam A., Ryu E. (2019). Instafamous and social media influencer marketing. Mark. Intell. Plan..

[19] Jin S.V., Ryu E. (2020). “I’ll buy what she’s #wearing”: The roles of envy toward and parasocial interaction with influencers in Instagram celebrity-based brand endorsement and social commerce. J. Retail. Consum. Serv..

[20] Jin S.V., Ryu E. (2019). Instagram fashionistas, luxury visual image strategies, and vanity. J. Prod. Brand. Manag..

[21] Jin S.V. (2018). Interactive effects of Instagram foodies’ hashtagged #foodporn and peer users’ eating disorder on eating intention, envy, parasocial interaction, and online friendship. CyberPsychol. Behav. Soc. Netw..

[22] Jin S.V., Ryu E., Muqaddam A. (2018). Dieting 2.0!: Moderating effects of Instagrammers’ body image and Instafame on other Instagrammers’ dieting intention. Comput. Hum. Behav..

[23] Schops J.D., Kogler S., Hemetsberger A. (2020). (De-)stabilizing the digitized fashion market on Instagram-dynamics of visual performative assemblages. Consum. Mark. Cult..

[24] Richins M.L., Dawson S. (1992). A consumer values orientation for materialism and its measurements: Scale development and validation. J. Consum. Res..

[25] Ozimek P., Forster J. (2017). The impact of self-regulatory states and traits on Facebook use: Priming materialism and social comparisons. Comput. Hum. Behav..

[26] Thoumrungroje A. (2014). The influence of social media intensity and EWOM on conspicuous consumption. Procedia. Soc. Behav. Sci..

[27] Lin R. (2018). Silver lining of envy on social media? The relationships between post content, envy type, and purchase intentions. Internet Res..

[28] Smith R.H., Kim S.H. (2007). Comprehending envy. Psychol. Bull..

[29] Belk R.W., Kinnear T.C. (1984). Three scales to measure constructs related to materialism: Reliability, validity, and relationships to measure happiness. Advances in Consumer Research.

[30] Vandenbroele J., Van Kerckhove A., Geuens M. (2020). If you work it, flaunt it: Conspicuous displays of exercise efforts increase mate value. J. Bus. Res..

[31] Markus H. (1977). Self-schemata and processing information about the self. J. Pers. Soc. Psychol..

[32] Ahadzadeh A.S., Sharif S.P., Ong F.S. (2017). Self-schema and self-discrepancy mediate the influence of Instagram usage on body image satisfaction among youth. Comput. Hum. Behav..

[33] Sinton M.M., Birch L.L. (2006). Individual and sociocultural influences on pre-adolescent girls’ appearance schemas and body dissatisfaction. J. Youth. Adolesc..

[34] Birkeland R., Thompson K., Herbozo S., Roehrig M., Cafri G., van den Berg P. (2005). Media exposure, mood, and body image dissatisfaction: An experimental test of person versus product priming. Body Image.

[35] Herbozo S., Thompson J.K. (2010). The effects of ambiguous appearance-related feedback on body image, mood states, and intentions to use body change strategies in college women: An experimental study. Body Image.

[36] Van den Berg P., Thompson J.K. (2007). Self-schema and social comparison explanations of body dissatisfaction: A laboratory investigation. Body Image.

[37] Higgins E.T. (1987). Self-discrepancy: A theory relating self and affect. Psychol. Rev..

[38] Richins M.L., Wallendorf M., Anderson P. (1987). Media, materialism, and human happiness. Advances in Consumer Research.

[39] Mandel N., Rucker D.D., Levav J., Galinsky A.D. (2017). The compensatory consumer behavior model: How self-discrepancies drive consumer behavior. J. Consum. Psychol..

[40] Dittmar H. (2005). A new look at compulsive buying: Self-discrepancies and materialistic values as predictors of compulsive buying tendency. J. Soc. Clin. Psychol..

[41] Gornik-Durose M.E., Pilch I. (2016). The dual nature of materialism. How personality shapes materialistic value orientation. J. Econ. Psychol..

[42] Ahuvia A.C., Wong N.Y. (2002). Personality and values-based materialism: Their relationship and origins. J. Consum. Psychol..

[43] Kasser T. (2002). The High Price of Materialism.

[44] Li J., Lu M., Xia T., Guo Y. (2018). Materialism as compensation for self-esteem among lower-class students. Pers. Individ. Differ..

[45] Blachnio A., Przepiorka A., Pantic I. (2016). Association between Facebook addiction, self-esteem, and life satisfaction: A cross-sectional study. Comp. Human Behavior..

[46] Okazaki S., Schuberth F., Tagashira T., Andrade V. (2021). Sneaking the dark side of brand engagement into Instagram: The dual theory of passion. J. Bus. Res..

[47] Sharp G., Fardouly J., Bromberg M., Leaver T., Gerrard Y. Instagram Can Make Teens Feel Bad About Their Body, But Parents Can Help. Here’s How. The Conversation, 23 September 2021. https://theconversation.com/instagram-can-make-teens-feel-bad-about-their-body-but-parents-can-help-heres-how-168093.

[48] Tiggemann M., Velissaris V.G. (2020). The effect of viewing challenging “reality check” Instagram comments on women’s body image. Body Image.

[49] Veldhuis J., Alleva J.M., Bij de Vaate A.J.D., Keijer M., Konijn E.A. (2020). Me, my selfie, and I: The relations between selfie-behaviors, body image, self-objectification, and self-esteem in young women. Psychol. Pop. Media Cult..

[50] Yellowlees R., Dingemans A.E., Veldhuis J., Bij de Vaate J.D. (2019). Face yourself(ie): Investigating selfie-behavior in females with severe eating disorder symptoms. Comput. Hum. Behav..

[51] Ghaznavi J., Taylor L.D. (2015). Bones, body parts, and sex appeal; An analysis of #thinspiration images on popular social media. Body Image.

[52] Tiggemann M., Zaccardo M. (2015). “Exercise to be fit, not skinny”: The effect of fitspiration imagery on women’s body image. Body Image.

[53] Fardouly J., Diedrichs P.C., Vartanian L.R., Halliwell E. (2015). Social comparisons on social media: The impact of Facebook on young women’s body image concerns and mood. Body Image.

[54] Fardouly J., Vartanian L.R. (2015). Negative comparisons about one’s appearance mediate the relationship between Facebook usage and body image concerns. Body Image.

[55] Arseniev-Koehler A., Lee H., McCormick T., Moreno M.A. (2016). #Proana: Pro-eating disorder socialization on Twitter. J. Adolesc. Health.

[56] Radovic A., Gmelin T., Stein B.D., Miller E. (2017). Depressed adolescents’ positive and negative use of social media. J. Adolesc..

[57] De Vries D.A., Peter J., de Graaf H., Nikken P. (2015). Adolescents’ social network site use, peer appearance-related feedback, and body dissatisfaction: Testing a mediation model. J. Youth Adolesc..

[58] Engeln R., Loach R., Imundo M.N., Zola A. (2020). Compared to Facebook, Instagram use causes more appearance comparison and lower body satisfaction in college women. Body Image.

[59] Vohs K.D., Bardone A.M., Joiner T.E., Abramson L.Y. (1999). Perfectionism, perceived weight status, and self-esteem interact to predict bulimic symptoms: A model of bulimic symptom development. J. Abnorm. Psychol..

[60] Saunders J.F., Eatonm A.A., Aguilar S. (2020). From self(ie)-objectification to self-empowerment: The meaning of selfies on social media in eating disorder discovery. Comput. Hum. Behav..

[61] Stice E., Shaw H.E. (2002). Role of body dissatisfaction in the onset and maintenance of eating pathology: A synthesis of research findings. J. Psychosom. Res..

[62] Bue A.C.C. (2020). The looking glass selfie: Instagram use frequency predicts visual attention to high-anxiety body regions in young women. Comput. Hum. Behav..

[63] Howard L.M., Heron K.E., MacIntyre R.I., Myers T.A., Everhard R.S. (2017). Is use of social networking sites associated with young women’s body dissatisfaction and disordered eating? A look at Black-White racial differences. Body Image.

[64] Mabe A.G., Forney K.J., Keel P.K. (2014). Do you “like” my photo? Facebook use maintains eating disorder risk. Int. J. Eat. Disord..

[65] Crusius J., Lange J. (2014). What catches the envious eye? Attentional biases within malicious and benign envy. J. Exp. Soc. Psychol..

[66] Arnocky S., Perilloux C., Cloud J.M., Bird B.M., Thomas K. (2015). Envy mediates the link between social comparison and appearance enhancement in women. Evol. Psychol. Sci..

[67] Chae J. (2017). Virtual makeover: Selfie-taking and social media use increase selfie-editing frequency through social comparison. Comput. Hum. Behav..

[68] Wang R., Yang F., Haigh M.M. (2017). Let me take a selfie: Exploring the psychological effects of posting and viewing selfies and groupies on social media. Telemat. Inform..

[69] Shin Y., Kim M., Im C., Chong S.C. (2017). Selfie and self: The effect of selfies on self-esteem and social sensitivity. Pers. Individ. Differ..

[70] Sabiniewicz A., Borkowska B., Serafinska K., Sorokowski P. (2017). Is love related to selfies? Romantic selfie posting behavior and love levels among women and men. Pers. Individ. Differ..

[71] Ma J.W., Yang Y., Wilson J.A.J. (2017). A window to the ideal self: A study of UK Twitter and Chinese Sina Weibo selfie-takers and the implications for marketers. J. Bus. Res..

[72] Sorokowski P., Sorokowska A., Oleszkiewicz A., Frackowiak T., Huk A., Pisanski K. (2015). Selfie-posting behaviors are associated with narcissism among men. Pers. Individ. Differ..

[73] Weiser E.B. (2015). #Me: Narcissism and its facets as predictors of selfie-posting frequency. Pers. Individ. Differ..

[74] Sorokowska A., Oleszkiewicz A., Frackowiak T., Pisanski K., Chmiel A., Sorokowski P. (2016). Selfies and Personality: Who Posts Self-Portrait Photographs?. Pers. Individ. Differ..

[75] Andreassen C.S., Pallesen S., Griffiths M.D. (2017). The relationship between addictive use of social media, narcissism and self-esteem: Findings from a large national survey. Addict. Behav..

[76] Morf C.C., Rhodewalt F. (2001). Unraveling the paradoxes of narcissism: A dynamic self-regulatory processing model. Psychol. Inq..

[77] Egan V., McCorkindale C. (2007). Narcissism, vanity, personality, and mating effort. Pers. Individ. Differ..

[78] Ha T., Overbeek G., Engels R.C.M.E. (2010). Effects of attractiveness and social status on dating desire in heterosexual adolescents: An experimental study. Arch. Sex. Behav..

[79] Gordon K.H., Dombeck J.J. (2010). The associations between two facets of narcissism and eating disorder symptoms. Eat. Behav..

[80] Back M.D., Schmukle S.C., Egloff B. (2010). Why are narcissists so charming at first sight? Decoding the narcissism-popularity link at zero acquaintance. J. Pers. Soc. Psychol..

[81] Smith M.M., Sherry S.B., Chen S., Saklofske D.H., Flett G.L., Hewitt P.L. (2016). Perfectionism and narcissism: A meta-analytic review. J. Res. Pers..

[82] Lemaitre B. (2016). Connecting the obesity and the narcissism epidemics. Med. Hypotheses.

[83] Krekels G., Pandelaere M. (2015). Dispositional greed. Pers. Individ. Differ..

[84] Pinkasavage E., Arigo D., Schumacher L.M. (2015). Social comparison, negative body image, and disordered eating behavior: The moderating role of coping style. Eat. Behav..

[85] Lin L., Soby M. (2016). Appearance comparisons styles and eating disordered symptoms in women. Eat. Behav..

[86] Thogersen-Ntoumani C., Ntoumanis N., Cumming J., Chatzisarantis N.L.D. (2011). When feeling attractive matters too much to women: A process underpinning the relation between psychological need satisfaction and unhealthy weight control behaviors. Motiv. Emot..

[87] Schleien J.L., Bardone-Cone A.M. (2016). Competitiveness as moderator of the relation between appearance-related factors and disordered eating behaviors. Body Image.

[88] Alcaraz-Ibanez M., Sicilia A., Diez-Fernandez D.M., Paterna A. (2020). Physical appearance comparisons and symptoms of disordered eating: The mediating role of social physique anxiety in Spanish adolescents. Body Image.

[89] Parrott W.G., Smith R.H. (1993). Distinguishing the experiences of envy and jealousy. J. Pers. Soc. Psychol..

[90] Lange J., Crusius J., Hagemeyer B. (2016). The evil queen’s dilemma: Linking narcissistic admiration and rivalry to benign and malicious envy. Eur. J. Pers..

[91] Luchner A.F., Houston J.M., Walker C., Houston A.M. (2011). Exploring the relationship between two forms of narcissism and competitiveness. Pers. Individ. Differ..

[92] Ohmann K., Burgmer P. (2016). Nothing compares to me: How narcissism shapes comparative thinking. Pers. Individ. Differ..

[93] Krizan Z., Johar O. (2012). Envy divides the two faces of narcissism. J. Pers..

[94] Lim M., Yang Y. (2015). Effects of users’ envy and shame on social comparison that occurs on social network services. Comput. Hum. Behav..

[95] Barnett M.D., Sharp K.J. (2017). Narcissism, gender, and evolutionary theory: The role of private and public self-absorption. Pers. Individ. Differ..

[96] Gilbert P., Price J., Allan S. (1995). Social comparison, social attractiveness, and evolution: How might they be related?. New Ideas Psychol..

[97] Festinger L. (1954). A theory of social comparison processes. Hum. Relat..

[98] Goncalves M.K., Campbell L. (2014). The dark triad and the derogation of mating competitors. Pers. Individ. Differ..

[99] Buss D.M., Schmitt D.P. (1993). Sexual strategies theory: An evolutionary perspective on human mating. Psychol. Rev..

[100] Hendrickse J., Arpan L.M., Clayton R.B., Ridgway J.L. (2017). Instagram and college women’s body image: Investigating the roles of appearance-related comparisons and intrasexual competition. Comput. Hum. Behav..

[101] Fisher M., Cox A. (2010). Four strategies used during intrasexual competition for mates. Pers. Relatsh..

[102] Bergman S.M., Fearrington M.E., Davenport S.W., Bergman J.Z. (2011). Millennials, narcissism, and social networking: What narcissists do on social networking sites and why. Pers. Individ. Differ..

[103] Heatherton T.F., Polivy J. (1991). Development and validation of a scale for measuring state self-esteem. J. Pers. Soc. Psychol..

[104] Ohanian R. (1990). Construction and validation of a scale to measure celebrity endorsers’ perceived expertise, trustworthiness, and attractiveness. J. Advert..

[105] Richins M.L. (2004). The material values scale: Measurement properties and development of a short form. J. Consum. Res..

[106] Rosenberg M. (1965). Society and the Adolescent Self-Image.

[107] Hill R.W., Huelsman T.J., Furr R.M.M., Kibler J., Vincente B.B., Kennedy C. (2004). A new measure of perfectionism. The perfectionism inventory. J. Pers. Assess..

[108] Friborg O., Clausen L., Rosenvinge J.H. (2013). A five-item screening version of the Eating Disorder Inventory (EDI-3). Compr. Psychiatry.

[109] Lange J., Crusius J. (2015). Dispositional envy revisited: Unraveling the motivational dynamics of benign and malicious envy. Pers. Soc. Psychol. Bull..

[110] Ellison N.B., Steinfield C., Lampe C. (2007). The benefits of Facebook ‘friends’: Social capital and college students’ use of online social network sites. J. Comput. Mediat. Comm..

[111] Rosenthal S.A., Hooley J.M., Steshenko Y. (2020). The narcissistic grandiosity scale: A measure to distinguish narcissistic grandiosity from high self-esteem. Assessment..

[112] Landolt M.A., Lalumiere M.L., Quinsey V.L. (1995). Sex differences in intra-sex variations in human mating tactics: An evolutionary approach. Ethol. Sociobiol..

[113] Faer L.M., Hendriks A., Abed R.T., Figueredo A.J. (2005). The evolutionary psychology of eating disorders: Female competition for mates or for status?. Psychol. Psychother.: Theory Res. Pract..

[114] Gibbons F.X., Buunk B.P. (1999). Individual differences in social comparison: Development of a scale of social comparison orientation. J. Pers. Soc. Psychol..

[115] Thompson J.K., Heinberg L., Tantleff S. (1991). The Physical Appearance Comparison Scale (PACS). Behav. Ther..

[116] Ajzen I. (1991). The theory of planned behavior. Organ. Behav. Hum. Decis. Process..

[117] APA Dictionary of Psychology: Heuristic Value. https://dictionary.apa.org/heuristic-value.

[118] Buss D.M. (2009). The great struggle of life: Darwin and the emergence of evolutionary psychology. Am. Psychol..

[119] Button E.J., Loan P., Davies J., Sonuga-Barke E.J.S. (1997). Self-esteem, eating problems, and psychological well-being in a cohort of schoolgirls aged 15–16: A questionnaire and interview study. Eat. Disor..

[120] Jin S.V., Ryu E., Muqaddam A. (2019). Romance 2.0 on Instagram! “What type of girlfriend would you date?”. Evol. Psychol..

[121] Trekels J., Ward L.M., Eggermont S. (2018). I “like” the way you look: How appearance-focused and overall Facebook use contribute to adolescents’ self-sexualization. Comput. Hum. Behav..

[122] Taylor D.G., Strutton D. (2016). Does Facebook usage lead to conspicuous consumption? The role of envy, narcissism, and self-promotion. J. Res. Interact. Mark..

[123] Ackerman R.A., Witt E.A., Donnellan M.B., Trzesniewski K.H., Robins R.W., Kashy D.A. (2011). What does the narcissistic personality inventory really measure?. Assessment.

